# Low frequency of *Plasmodium falciparum hrp2/3* deletions from symptomatic infections at a primary healthcare facility in Kilifi, Kenya

**DOI:** 10.3389/fepid.2023.1083114

**Published:** 2023-02-21

**Authors:** Dorcas Okanda, Leonard Ndwiga, Victor Osoti, Nicole Achieng, Juliana Wambua, Caroline Ngetsa, Peter Lubell-Doughtie, Anuraj Shankar, Philip Bejon, Lynette Isabella Ochola-Oyier

**Affiliations:** ^1^ Biosciences Department, KEMRI-Wellcome Trust Research Programme, Kilifi, Kenya; ^2^ ONA Systems Inc, Burlington, VT, United States; ^3^ Nuffield Department of Medicine, Centre for Clinical Vaccinology and Tropical Medicine, Churchill Hospital, University of Oxford, Oxford, United Kingdom

**Keywords:** *Pfhrp2*, *Pfhrp3*, gene deletions, malaria RDTs, *Plasmodium falciparum*

## Abstract

There is a growing concern for malaria control in the Horn of Africa region due to the spread and rise in the frequency of *Plasmodium falciparum* Histidine-rich Protein (hrp) 2 and 3 deletions. Parasites containing these gene deletions escape detection by the major PfHRP2-based rapid diagnostic test. In this study, the presence of *Pfhrp2/3* deletions was examined in uncomplicated malaria patients in Kilifi County, from a region of moderate-high malaria transmission. 345 samples were collected from the Pingilikani dispensary in 2019/2020 during routine malaria care for patients attending this primary health care facility. The Carestart™ RDT and microscopy were used to test for malaria. In addition, qPCR was used to confirm the presence of parasites. In total, 249 individuals tested positive for malaria by RDT, 242 by qPCR, and 170 by microscopy. 11 samples that were RDT-negative and microscopy positive and 25 samples that were qPCR-positive and RDT-negative were considered false negative tests and were examined further for *Pfhrp2/3* deletions. *Pfhrp2/3*-negative PCR samples were further genotyped at the dihydrofolate reductase (*Pfdhfr*) gene which served to further confirm that parasite DNA was present in the samples. The 242 qPCR-positive samples (confirmed the presence of DNA) were also selected for *Pfhrp2/3* genotyping. To determine the frequency of false negative results in low parasitemia samples, the RDT- and qPCR-negative samples were genotyped for *Pfdhfr* before testing for *Pfhrp2/3*. There were no *Pfhrp2* and *Pfhrp3* negative but positive for *dhfr* parasites in the 11 (RDT negative and microscopy positive) and 25 samples (qPCR-positive and RDT-negative). In the larger qPCR-positive sample set, only 5 samples (2.1%) were negative for both *hrp2* and *hrp3*, but positive for *dhfr*. Of the 5 samples, there were 4 with more than 100 parasites/µl, suggesting true *hrp2/3* deletions. These findings revealed that there is currently a low prevalence of *Pfhrp2* and *Pfhrp3* deletions in the health facility in Kilifi. However, routine monitoring in other primary health care facilities across the different malaria endemicities in Kenya is urgently required to ensure appropriate use of malaria RDTs.

## Introduction

The 2021 Malaria indicator survey highlights that the disease still presents a significant burden in Kenya, with about 70% of the population being at risk of infection. Malaria accounts for up to 15% of in-patient consultations in the country and individuals in endemic areas are at the greatest risk of disease (1). This is the case for most countries in sub-Saharan Africa, which carries the greatest burden of malaria globally (2). In Kenya, malaria transmission is heterogenous and varies based on factors such as altitude, temperature, and rainfall patterns. Malaria control mainly depends on effective diagnosis and treatment. The Kenyan National Malaria Control Program recommends the use of Microscopy and rapid diagnostic tests (RDTs) as first line modalities for malaria diagnosis (3). Microscopy plays a pivotal role in providing parasite density data for accurate diagnosis of disease. However, the process requires well trained personnel, adequate space, availability of quality microscopes, consistent power supply and laboratory resources and it has a relatively long turnaround time, a luxury that remote and resource limited settings might not afford. On the other hand, malaria RDTs have proven to be an effective and time-saving tool for diagnosis as they are used as a point of care test, with prompt delivery of results (results can be obtained in 15–20 min) (4). The World Health Organization also recommends the use of malaria RDTs as convenient alternatives for diagnosis in malaria endemic regions, particularly in Africa (5).

Malaria RDTs contain antibodies against *Plasmodium* antigens, including lactose dehydrogenase, aldolase, and the histidine rich protein 2 (HRP2). However, the HRP2-based RDTs are the most preferred for their ability to uniquely detect infection with *Plasmodium falciparum*, the *Plasmodium* species that causes the greatest burden of malaria (6). They have also been shown to be more sensitive and heat stable, compared to the other commercially available RDTs (7, 8). While HRP2-based RDTs are primarily designed to detect the HRP2 antigen, they are also able to detect its isoform, HRP3, which has high sequence similarity to HRP2, jointly contributing to RDT test results (9). These RDTs have been instrumental in most malaria endemic areas, where accurate diagnosis is vital for effective control of the disease. The HRP2 protein is 60–105 kDa in size, water-soluble and abundantly expressed by the asexual stages of *Plasmodium falciparum* (10). Nonetheless, its main function in the parasite remains largely unclear.

Deletions in *Plasmodium falciparum* hrp2/3 (*Pfhrp2/3*) genes, compromise the quality of patient care as they result in false negative tests. In addition, they present a significant setback to malaria control because the parasite escapes detection, limiting effective diagnosis and treatment. Deletions in *Pfhrp2/3* were originally reported in Peru (4) and have since been documented at relatively high frequencies in other South American countries such as Suriname (11), Brazil (12, 13) and Colombia (14). In Africa, *Pfhrp2/3* deletions have been reported at fairly low frequencies of <5%, except in Ethiopia and Eritrea where proportions of up to 80% have been documented, leading to policy changes in the use RDTs based on these proteins (9, 15–21). Nonetheless, the lack of a uniform denominator for calculating these frequencies, limits the accurate interpretation of deletion data and comparison of results across regions (22, 23). The World Health Organization (WHO) recommends that use of HRP2/3 based RDTs be discontinued in regions where these deletions exceed 5% (24). While RDTs remain effective in most African countries, including Kenya, routine surveillance for possible mutations is important for policy makers as it guides appropriate use of these first-line diagnostic tools.

In Kenya, limited data exist on the status of *Pfhrp2/3* deletions, although the RDTs in current use are still effective. Routine data on the status of these deletions would however benefit national malaria control programs by guiding policies on appropriate RDT use. The prevalence of *Pfhrp2/3* deletions in the south of Kilifi County, a moderate to high malaria transmission setting in Kenya was first determined based on the WHO recommendations of a RDT result and secondly from a criterion set in this study of a real-time PCR test result, which also included a cycle threshold (Ct) cut-off to increase the sensitivity of detecting parasite DNA. Although the *Pfhrp2/3* deletions observed occurred at relatively low frequencies, these data provide the baseline for regular surveillance of malaria RDTs used in the country.

## Materials and methods

### Study area and sample selection

**Figure 1:**
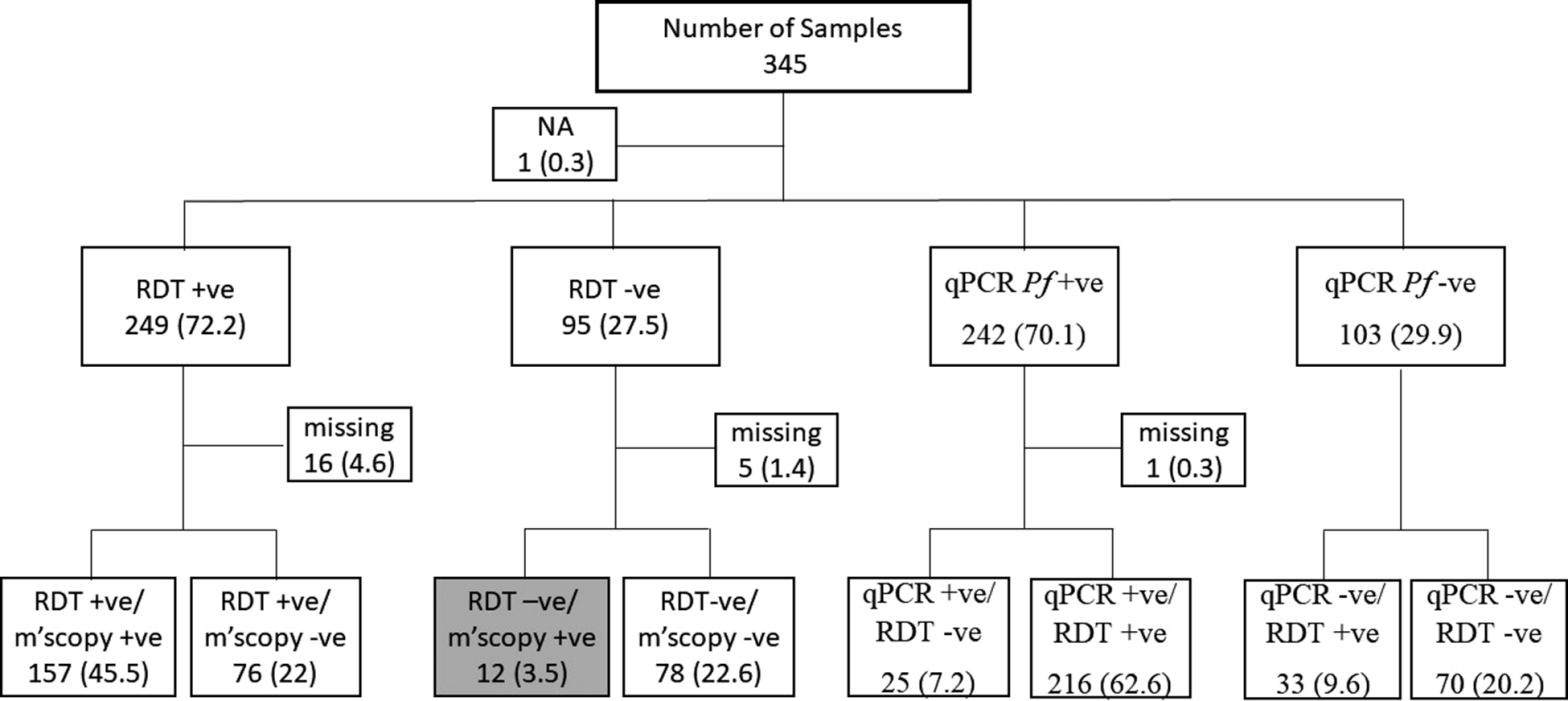
The sample selection criteria for the study. Samples were selected based on RDT the routine malaria diagnostic test conducted at health facilities. Thus, 12 discordant samples were RDT negative but positive by microscopy (grey box). Sample selection was also based on qPCR results following a routine assessment done within this study. NA, sample with no RDT data; Missing, samples with RDT but no microscopy data or qPCR but no RDT data. The values in brackets represent the frequencies of the respective categories relative to the overall sample size of 345.

EDTA blood samples from 345 patients between November 2019 and February 2020 were obtained from a malaria monitoring study that aimed to compare the performance of two malaria RDTs used for diagnosis in Pingilikani dispensary (25). Pingilikani is located within the Kilifi Health and Demographic Surveillance System (KHDSS), approximately 30 km south of Kilifi town (26). The malaria monitoring study (approved by the ethics review committee of the Kenya Medical Research Institute under protocol number SERU 2617) routinely consents patients with malaria for a dried blood spot (DBS) and EDTA blood sample alongside the diagnostic Carestart™ RDT and a microscopy test. The microscopy was conducted in the KEMRI-Wellcome Trust Research Programme GCLP accredited labs by competent expert microscopists, as per the WHO criteria, who routinely support clinical trials. The lab is also registered for quantitative Polymerase Chain Reaction (qPCR), UKNEQAS. The qPCR assay targeted the *P. falciparum* 18S ribosomal DNA. The 345 samples were categorized as either RDT and microscopy positive, RDT positive and microscopy negative, RDT and microscopy negative, or RDT negative and microscopy positive. The latter category (RDT negative and microscopy positive) was of interest as it highlighted a discordant sample set that were suspected false negatives, hence explored further for *Pfhrp2* and *hrp3* gene deletions, the primary targets in the Carestart™ RDT. Within the broader sample set, we explored the utility of using clinical surveillance samples for routine molecular monitoring of *Pfhrp2* and *Pfhrp3* gene deletions. Since the samples had been analyzed by qPCR alongside RDT for clinical diagnosis, all the samples positive by qPCR were genotyped for the *Pfhrp2/3* gene deletions along with 30% of the qPCR negative samples. The latter samples were first genotyped for *Pfdhfr* to confirm parasite DNA quality and those that tested positive were subjected to *hrp2/3* PCRs. We also analyzed a subset of 25 samples that were positive by qPCR but negative by RDT and 23 samples that were positive by RDT but negative by both qPCR and microscopy (to determine the rate of detecting false negative results at very low parasitemia). The sample selection criteria based on RDT and qPCR are shown in Figure 1.

### DNA extraction and *Pfhrp2/3* PCR

Following the manufacturer's instructions, parasite genomic DNA was extracted from the EDTA whole blood samples using the QIAamp® DNA Blood Mini kit (QIAGEN). The QIAcube HT (QIAGEN) platform was used for automated extraction, and the Nanodrop, ND 1000 Spectrophotometer was used to assess the concentration and purity (A260/A280 ratios) of the isolated genomic DNA. To create working concentrations of 5 ng/µl that could be used in the following procedures, the extracted DNA samples were diluted in sterile, double-distilled water and aliquots stored at −20°C. The *Pfhrp2/3* PCRs were conducted in a semi-nested approach, using previously published primers (targeting Exon 2 of both genes) (17) listed in Table 1. The *Plasmodium falciparum* dhfr gene was also included in the PCRs, to confirm the presence and quality of parasite DNA in samples with possible *Pfhrp2/3* deletions. The Expand High Fidelity PCR system (Roche™) was used to conduct conventional PCR. The annealing temperature for each primer combination (Supplementary Table S1) was determined through gradient PCR, conducted using *Pf*3D7 DNA as the template. Given that the *Pfhrp2* and *Pfhrp3* PCRs were semi-nested, the first round of amplification was conducted using the F1&R1 primer combination for each gene. Amplicons from the first amplification round were then used as templates for the second PCR which was performed using the F2&R1 primer combinations. A single reaction volume of 10.5 µl consisted of 1μl of template DNA (5 ng/μl), 6.56 µl of ultrapure DNase/RNase free distilled water, 0.2 µl of 10 mM deoxynucleotides triphosphate (dNTPs), 1 µl of 25 mM MgCl_2_, 0.3 µl of 10pmol/µl forward primer (F1or F2), 0.3 µl of 10pmol/µl reverse primer, 1 µl of 10X PCR buffer with 15 mM MgCl_2_ and 0.14 µl of 3.5 U/µl Expand High fidelity PCR Taq polymerase (Roche). The thermal cycling conditions for each round of PCR were 94°C for 2 min, 10 cycles of 94°C for 15 s, 54°C - 30 s and 72°C for 2 min, 25 cycles of 94°C for 15 s, 54- 30 s and 72°C for 2 min then a final extension at 72°C for 2 min. The positive controls were DNA from the *P. falciparum* 3D7 (with both *Pfhrp2* and *Pfhrp3*), Dd2 (*Pfhrp2* deleted) and HB3 (*Pfhrp3* deleted) laboratory isolates. The *Pfdhfr* PCRs were performed using published primers (27) in a 10.5 µl reaction (*dhfr* had one forward primer, unlike *hrp2/3* which had two, each). The thermal cycling conditions included 94°C for 2 min, 10 cycles of 94°C for 15 s, 58°C for 30 s and 68°C for 2 min, 25 cycles of 94°C for 15 s, 58- 30 s and 68°C for 2 min then a final extension at 72°C for 2 min. *P. falciparum* 3D7 DNA was similarly used as the positive control. Nuclease-free water was used as the template for the negative controls, for all three genes. 2% (w/v) agarose gels stained with 5 µl of 20,000X RedSafe DNA staining solution, were used to visualize amplification products. 5 µl of each PCR product was used for gel electrophoresis. All gels were run at 100 V for 40 min then imaged using the Bio-Rad ChemiDoc XRS + TM imaging system.

**Table 1:** A summary of the *Pfhrp2*, *Pfhrp3* and *pfdhfr* genotyping results from different sample categories.

Genotypes	RDT−ve/m'scopy+ve (*n* = 11)	qPCR+ve/RDT−ve (*n* = 25)	qPCR+ve/RDT−ve and Ct ≤ 34 (*n* = 15)	qPCR+ve (*n* = 242)	qPCR+ve and Ct ≤ 34 (*n* = 203)	qPCR−ve and *Pfdhfr*+ve (*n* = 48)	RDT+ve/qPCR and microscopy−ve (*n* = 23)
*Pfhrp2*+ve/Pfh*rp3*+ve	3 (3.2)	11 (4.5)	8 (3.3)	153 (63.2)	139 (57.4)	5 (4.9)	2 (0.8)
*Pfhrp2*+ve/Pfh*rp3*−ve	3 (3.2)	9 (3.7)	3 (1.2)	27 (11.1)	11 (4.5)	8 (7.8)	2 (0.8)
*Pfhrp2*−ve/Pfh*rp3*+ve	4 (4.2)	4 (1.6)	4 (1.6)	24 (9.9)	24 (9.9)	1 (0.97)	1 (0.4)
*Pfhrp2*−ve/Pf*hrp3*−ve (Pf*dhfr*+ve)	0	0	0	6 (2.5)	5 (2.1)	1 (0.97)^a^	0
*Pfhrp2*−ve/Pf*hrp3*−ve (Pf*dhfr*−ve)	1 (1.1)	1 (0.4)^b^	0	4 (1.7)^c^	0	0	0

The numbers in () are %. There were 28 and 24 samples missing from the qPCR+ve and qPCR+ve and Ct ≤ 34 columns respectively, hence the samples were not genotyped for either *Pfhrp2* or *Pfhrp3*. For the qPCR−ve column, from the 48 selected samples 15 were *Pfdhfr* positive while the remaining samples were negative. In the RDT+ve/qPCR and Microscopy−ve column, only 5 were *Pfdhfr* positive and the rest of the samples were negative.

^a^ This sample was negative by qPCR and microscopy and thus a false positive.

^b^ The sample was negative by qPCR (Ct = 40) and microscopy, all 3 PCRs confirm the results and based on the stringent Ct cut-off of ≤34, the samples do not meet the criteria of a true result.

^c^ The samples were all negative by qPCR (Cts ≥ 36) and microscopy, the 3 PCRs confirm the results and based on the stringent Ct cut-off of ≤34, the samples do not meet the criteria of true result.

### *P. falciparum* qPCR analysis

Real-time amplification for detection and absolute detection of *P. falciparum* were performed on the ABI 7500 Real-Time PCR system (Applied Biosystems) as previously described (28) using TaqMan based *P. falciparum* specific probes. The reaction was performed in a final volume of 25 μl containing: 2.5 µl each of *P. falciparum* 18S rDNA forward and reverse primers (10 pmol/µl), 0.625 µl of 18S probe (10 pmol/µl), 12.5 µl TaqMan universal PCR master mix (2X), 6.75 µl of sample or 3D7 control samples, with the remaining volume PCR clean water. The following RT-PCR conditions were used: 50°C for 2 min, 95°C for 10 min, and then 45 cycles of 95°C for 15 s and 60°C for 1 min. All qPCR assays were run with appropriate controls including the Non-Template Control (NTC). For parasite quantification, eight defined standards with varying parasitemias were included, Standard 1: 460,000 parasites/μl, Standard 2: 27,000 parasites/μl, Standard 3: 15,000 parasites/μl, Standard 4: 10,000, Standard 5: 100 parasites/μl, Standard 6: 10 parasites/μl, Standard 7: 1 parasites/μl and Standard 8: 0.1 parasites/μl. Following an extrapolation from the standard curve, samples with a Ct value of above 34 were considered *P. falciparum* negative (<5 parasites/ul).

### Sequencing and sequence analysis

We sequenced samples that were *Pfhrp2* and *Pfhrp3* positive, from the 12 that were microscopy positive but RDT negative. Following PCR, amplicons were purified using the ExoSap-IT™ (Affymetrix™) reagent, then sequenced using the BigDye® Terminator v3.1 cycle sequencing kit from Applied Biosystems, UK. Sequencing was conducted using the same primers utilized for PCR. Briefly, a single reaction volume of 10 µl consisted of 2 µl, purified template DNA, 0.5 µl BigDye® Terminator enzyme mix, 1.75 µl 5X BigDye® sequencing buffer, 4.75 µl ultrapure DNase/RNase-free distilled water and 1 µl of 5pmol/µl sequencing primer. The cycling conditions for sequencing were as follows; an initial denaturation at 95°C for 30 s, 25 cycles of 96°C for 10 s, 50°C for 5 s and 60°C for 4 min, then a final hold at 15°C for 10 min. The products of sequencing were thereafter subjected to ethanol precipitation then capillary electrophoresis on the 3730xl DNA analyser (ILRI, Kenya), to generate sequence chromatograms. Finally, sequence analysis was conducted using CLC Workbench Version 7.7.1 (QIAGEN).

### Statistical analysis

Statistical analyses were conducted using Version 4.2.1 of the R software (29), where frequencies were calculated. A log_10_ transformation of parasitaemia was applied and used to compare the different *Pfhrp*2/3 genotypes using a Student's *t*-test with the statistical significance set at a *p* value <0.05. Box plots were used to show the 25th percentiles, geometric mean and 75th percentiles of the log transformed parasitemia.

## Results

### Semi-nested PCR amplification of false RDT negative samples based on microscopy

In this study, DNA from 345 samples tested for malaria by RDT, microscopy and qPCR were used and 249 (72.2%), 170 (49.3%) and 242 (70.1%) samples were positive for each test, respectively. There were 12 (3.47%) discordant samples that were *P. falciparum* negative by RDT but positive by microscopy (Figure 1), considered as false negative RDTs, were genotyped for *Pfhrp2* and *Pfhrp3*. *Pfdhfr* was used as a secondary gene to confirm the presence of parasite DNA in samples that tested negative for both *Pfhrp2* and *Pfhrp3*. One of the 12 discordant samples was missing and was therefore excluded from subsequent analysis. Among the remaining 11 samples, 3 (0.87%) tested positive for both *Pfhrp2* and *Pfhrp3*, 3 (0.87%) were positive for *Pfhrp2* but negative for *Pfhrp3*, while 4 (1.16%) were negative for *Pfhrp2* but positive for *Pfhrp3* (Table 1). Six of these samples had low parasitemias of <1,000 parasites/µl and the remaining sample was negative for all the genes tested (*Pfhrp2*, *Pfhrp3* and *Pfdhfr*). Notably, this sample was negative by qPCR but contained 3,520 parasites/µl.

### Semi-nested PCR amplification of false negative RDT samples based on qPCR

There were 25 (7.25%) samples that were positive by qPCR but negative by RDT, considered as discordant based on the qPCR test, and these were assessed for *Pfhrp2* and *Pfhrp3* deletions. Within this sample set, 6 samples were also microscopy positive. The results obtained, identified 11 (3.19%) samples that were positive for both *Pfhrp2* and *Pfhrp3*, 9 (2.61%) were positive for *pfhrp2* but negative for *Pfhrp3*, while 4 (1.16%) were positive for *pfhrp3* but negative for *Pfhrp2*. None of the samples were negative for both *Pfhrp2* and *Pfhrp3* but positive for *dhfr* (Table 1). We extended the analysis further and grouped the samples based on a Cycle threshold (Ct) cut-off of 34, which represented at least 5 parasites/µl (an extrapolation from our standard curve). This stringent cut-off yielded 17 discordant samples, from which the *Pfhrp3* negative only samples reduced to 4 (1.16%) with no change to the number of to the *Pfhrp2* negative only samples. There were no samples that tested negative for both *hrp2* and *hrp3* but positive for *dhfr*. In addition, the lone sample that was *Pfhrp2*−ve/Pf*hrp3*−ve (Pf*dhfr*−ve) but qPCR positive when Ct values were not considered as a criteria was further confirmed as lacking parasite DNA and dropped off with the stringent Ct cut-off.

### *Pfhrp2/3* genotyping results from qPCR positive samples

In addition to the discordant samples above, the analysis included a larger sample set that were *P. falciparum* positive by qPCR. A total of 242 samples were qPCR positive, the large majority, 44.4% were positive for both *hrp2* and *hrp3*, while only *6* (1.74%) were negative for both genes but positive for *Pfdhfr*. The remaining samples were either *Pfhrp2* negative only, *Pfhrp3* negative only, negative for all three genes or missing with no genotyping data. The stringent cut-off of Ct ≤ 34 resulted in 218 samples that met the criteria of a parasite positive sample. Similar to the qPCR discordant samples, there was reduction by about half [13, (4.1%)] in the numbers of *Pfhrp2* negative only samples. Only 1 sample was not accurately determined as *Pfhrp2* and Pf*hrp3* negative plus *Pfdhfr* positive, this sample was also microscopy negative. The four samples that were *Pfhrp2*−ve/Pf*hrp3*−ve (Pf*dhfr*−ve) were further confirmed as parasite negative samples and dropped off with the stringent Ct cut-off.

### *Pfhrp2/3* genotyping results from qPCR negative samples

As a final confirmation of the variation in qPCR and PCR specificity, 48 (∼47%) samples that were negative for *P. falciparum* by qPCR were selected and tested for the *Pfhrp2* and *Pfhrp3* deletions. All the 48 samples were genotyped for *Pfdhfr* to confirm that parasite DNA was present, yielding 15 positive samples, 69% of which were microscopy negative. Only 1 (0.29%) sample was negative for both genes but positive for *Pfdhfr* and further examination of the sample revealed that it was negative by microscopy and thus a false positive. Most, 8 (2.3%), of the remaining samples were negative for *Pfhrp3* only and only 2 of these samples were microscopy positive; and within the 5 *Pfhrp2* and *Pfhrp3* positive samples, 3 were microscopy positive.

### *Pfhrp2/3* genotyping results from RDT positive but microscopy and qPCR-negative samples

We had an additional category of samples (23) that were positive for *P. falciparum* by RDT, but negative by both microscopy and qPCR. Like the qPCR negative group, the samples were genotyped at the *dhfr* locus to select positive DNA samples for *Pfhrp2* and *Pfhrp3* testing. Of the 23, only 5 (1.45%) were positive for *dhfr* and of the 5, *Pfhrp2/3* data was obtained from 4 samples. Two (0.6%) of the four samples was positive for both *hrp2 and hrp3*, 2 (0.6%) were *Pfhrp3* negative only, while 1 (0.3%) was *Pfhrp2* negative only. None of the samples was negative for both *Pfhrp2* and *Pfhrp3*.

### Effect of parasitemia on *Pfhrp2/3* genotyping

**Figure 2:**
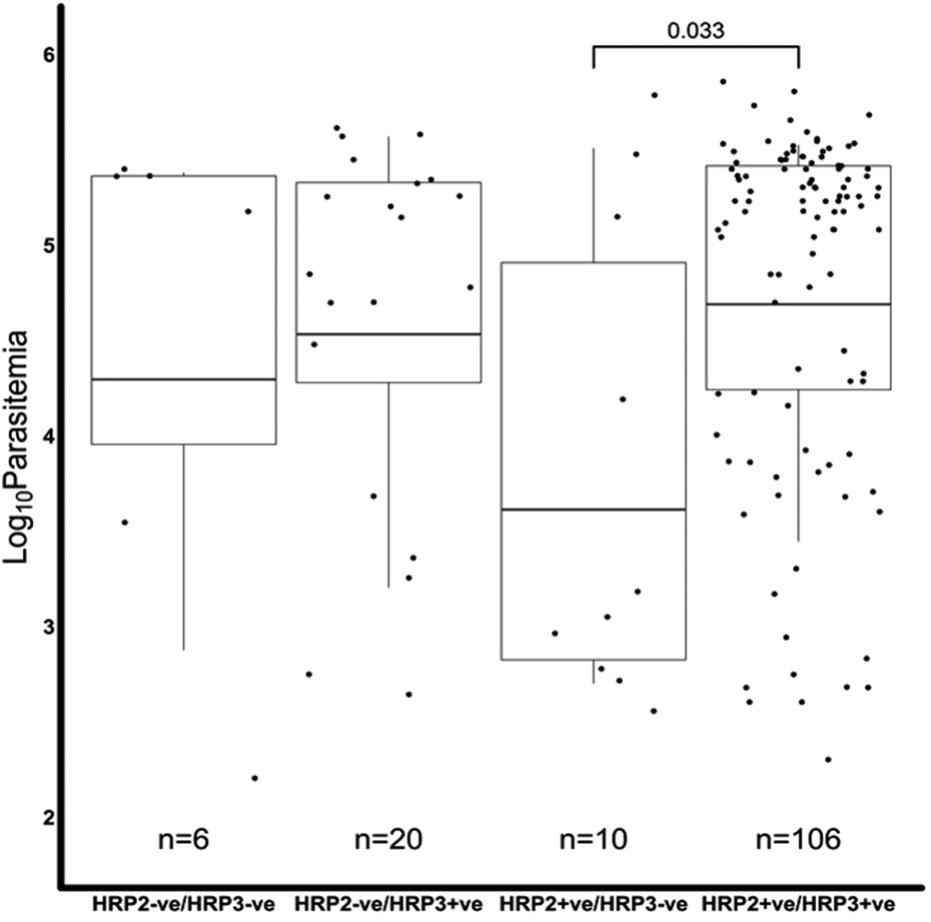
A comparison of the microscopy log base 10 parasitemia of the Pfhrp2/3 genotypes. The log_10_ geometric mean of the Pfhrp2/3 genotypes was, 4.30 (32,212 parasites/µl), 4.53 (44,247 parasites/µl), 3.61 (6,088 parasites/µl) and 4.69 (61,965 parasites/µl) for hrp2−ve/hrp3−ve, hrp2−ve/hrp3 + ve, hrp2 + ve/hrp3−ve and hrp2 + ve/hrp3 + ve, respectively. The hrp2 + ve/hrp3−ve group had significantly lower levels of parasitemia than the hrp2 + ve/hrp3 + ve category (*t*-test *p* < 0.05), in the overall sample set. There was no difference (*p* > 0.05) in parasitemia among the other hrp2/3 genotypes.

**Figure 3:**
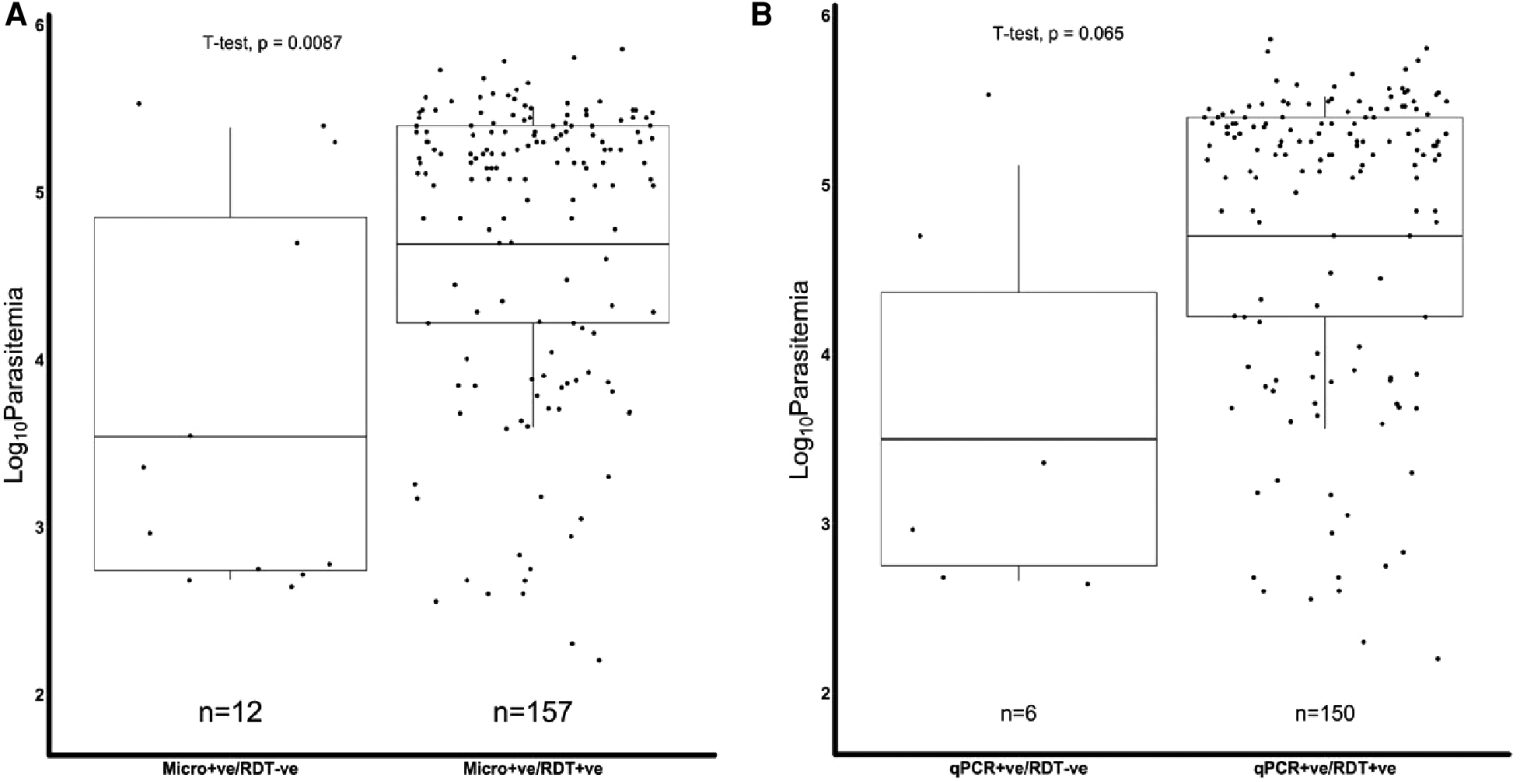
Combined Box and scatter diagrams comparing the microscopy log10 parasite densities of discordant and non-discordant samples. Horizontal lines represent the geometric means of log_10_ parasitemia. (**A**) Microscopy positives where the geometric mean parasitemia (log 10 parasitemia) was 3.5 (4978.0 parasites/μl) for m'scopy + ve/RDT−ve and 4.7 (61309.7 parasites/μl) for m'scopy + ve/RDT + ve. (**B**) qPCR positives, where the geometric mean parasitemia (log 10 parasitemia) was 3.5 (4427.3 parasites/μl) for qPCR + ve/RDT−ve and 4.7 (62589.9 parasites/μl) for qPCR + ve/RDT + ve.

We further assessed if the level of parasitemia influenced whether a sample tested positive or negative for *Pfhrp2*, *Pfhrp3* or both. There were 231 samples with *Pfhrp2/3* genotypes out of the total 345 samples. The geometric mean parasitemia of the respective *Pfhrp2/3* genotypes was 61,965 parasites/μl (*Pfhrp2/3 *+ ve), 6,088 parasites/μl (*Pfhrp2 *+ ve, *Pfhrp3*−ve), 44,247 parasites/μl (*Pfhrp2*−ve, *Pfhrp3 *+ ve), and 32,212 parasites/μl (*Pfhrp*2/3−ve). Overall, the analysis revealed that the *Pfhrp2 and Pfhrp3* negative and *Pfhrp2*−ve only samples had similar levels of parasitemia as those that tested positive for both genes (Figure 2). However, there was a significant difference in parasitemia between the *Pfhrp3*−ve only and *Pfhrp2 *+ ve/*hrp3 *+ ve samples (*p* = 0.033, Figure 2). Parasitemia levels were generally lower between samples that were discordant by either microscopy or qPCR and those that were non-discordant. Samples that were microscopy positive, but RDT negative had significantly (*p* = 0.0087) lower levels of parasitemia compared to those that were both microscopy and RDT-positive (Figure 3A), though cognizance is taken of the expected low numbers of discordant samples. There were 25 qPCR + ve/RDT−ve samples in total, however only 6 of these had microscopy data, and only 150 of the 216 qPCR + ve/RDT + ve samples had microscopy data. The low numbers of qPCR + ve/RDT−ve samples are likely to have attributed to the lack of significance observed in the comparison with qPCR + ve/RDT + ve samples (*p* = 0.065, Figure 3B).

### *Pfhrp2/3* sequencing

We sequenced 12 and 13 samples for *Pfhrp3* and *Pfhrp2*, respectively. The 7 and 9 good quality sequences obtained for *Pfhrp2* and *Pfhrp3* respectively, were subsequently analyzed for the presence of 3-, 6- and 9-amino acid repeats. A total of 20 repeat types were identified. Type 4 (AHH) and type 7 (AHHAAD) repeats were common in *Pfhrp2* and *Pfhrp3* and an additional 8 repeat types were described for *Pfhrp2*, while 5 more repeats were defined for *Pfhrp3* (Supplementary Table S2). Thus, demonstrating the similarity between both proteins and with the global population (30, 31).

## Discussion

This study was conducted in a primary healthcare facility that routinely conducts RDT tests to diagnose malaria. It had the advantage of examining microscopy and qPCR that were also routinely conducted for all the malaria positive samples presenting to the clinic. This provided a unique setting to interrogate *Pfhrp2* and *Pfhrp3* gene deletion frequencies, given the current expected low prevalence of the mutations in Kenya (21, 32). *Pfhrp2* and *Pfhrp3* deletions were therefore examined within this context of routine malaria monitoring in a clinical set up to detect uncomplicated malaria cases. Thus, malaria RDTs was the baseline selection criteria to initially identify discordant malaria test results when compared to microscopy and qPCR data.

The first observation was the lack of *Pfhrp2*−ve and *Pfhrp3*−ve parasites and the low (<5%) presence of *Pfhrp2*−ve only and *Pfhrp3*−ve only parasites within the RDT + ve and microscopy−ve discordant sample set. These samples were also significantly lower in parasitemia compared to the RDT + ve and microscopy + ve category, highlighting the difficulty in obtaining a true negative PCR result for either *Pfhrp2* or *Pfhrp3* and a true positive for the single copy confirmatory gene, *Pfdhfr*. This was also demonstrated by the lone sample that was qPCR negative, *Pfhrp2* and *Pfhrp3* semi-nested-PCR negative and *Pfdhfr* negative, albeit with a parasitemia of about 3,000 parasites/µl by microscopy. Thus, raising concerns on the specificity of the semi-nested-PCR approach used to determine *Pfhrp2* and *Pfhrp3* gene deletion frequencies.

The second analysis selected samples based on qPCR positivity and microscopy negativity; this approach doubled the discordant sample size and only 6 of the 25 samples were detectable by microscopy, indicating the increased sensitivity of qPCR in detecting samples with parasites. This superiority of qPCR over RDTs has been described before (33, 34). Despite the increased discordant sample size, no *Pfhrp2*−ve*/Pfhrp3*−ve parasites were detected and the frequency of *Pfhrp2*−ve only and *Pfhrp3*−ve only parasites decreased to <2%. The qPCR assay had the added advantage of excluding very low parasitemia levels that would be within the limit of detection of a true negative result. The combined Ct stringency cut-off improved the confidence in specificity of the semi-nested-PCR approach that determined the *Pfhrp2* and *Pfhrp3* gene deletion frequencies.

Thirdly, qPCR positivity as the first analysis served as a confirmatory test for the presence of parasite DNA in the samples, providing a much larger sample size. Thus, *Pfhrp2* and *Pfhrp3* gene deletion frequencies were examined without the need for *Pfdhfr* though it was still genotyped in samples that were *Pfhrp2*−ve and *Pfhrp3*−ve. The inclusion of the Ct cut-off increased the frequency of the *Pfhrp2*−ve only parasites to a more reliable frequency of about 5%, 10% for *Pfhrp3*−ve only parasites and 2% for *Pfhrp2*−ve and *Pfhrp3*−ve parasites.

Since qPCR−ve but *Pfdhfr* + ve samples may be either RDT + ve or microscopy + ve or both, they were also explored for *Pfhrp2* and *Pfhrp3* gene deletions. The only reliable results were the two *Pfhrp3*−ve only samples with microscopy data. Therefore, these analyses demonstrate the need for combined data to improve the accuracy of determining a true negative result.

The final analyses examined the potential to detect *Pfhrp2* and *Pfhrp3* false negatives using RDT + ve, qPCR−ve and microscopy−ve samples. This selection criteria represents samples that potentially had residual HRP2 and HRP3 in the blood from a cleared infection, since HRP2 can be found in blood up to 40 days following treatment (35). Thus, any amplified sample is likely due to DNA from dead parasites, sub-microscopic infections or infections below the qPCR detection limit. The false negative results for *Pfhrp2*−ve only and *Pfhrp3*−ve only samples were <1%.

It is apparent that the *Pfhrp2*−ve and *Pfhrp3*−ve parasites are detected in samples with low parasitemia and a larger sample is required to confirm this finding. However, the low parasitemia increases the chances of detecting false negative gene deletion results. The addition of qPCR provided confidence in detecting a true negative result and considerations need to be made when considering RDT positive and microscopy negative samples alone as they can contribute to the number of false negative results if they are not also determined to be qPCR negative, which is overall a more sensitive assay. Furthermore, from this sample size of 345, over a 4-month period, which is close to the current WHO *hrp2/3* protocol of 370 per health facility, the RDT positive and microscopy negative criteria and the qPCR positive and RDT negative discordant pairs did not detect any *Pfhrp2*−ve and *Pfhrp3*−ve parasites. This is expected considering the absence of the dual gene deletions in 192 febrile individuals studied in 2016 from a health facility in Busia county, Western Kenya (32) and in Mbita, Homa Bay County, where the prevalence of *Pfhrp2* deleted parasites was 9% from 89 samples analyzed from a sample size of 274 asymptomatic individuals in 2014 (36).

Similar to the earliest *Pfhrp2/3* study (36) conducted in Kenya, qPCR works well to define the sample selection criteria for *Pfhrp2/3* gene deletion screening in low prevalence settings. From this study, most parasites were *Pfhrp3* negative only at frequency of 10%, followed *Pfhrp2* negative only parasites at 5%, and *Pfhrp2* and *Pfhrp3* negative parasites were at a frequency of 2% from sample size of 345 febrile individuals. The higher prevalence of *Pfhrp3* negative only infections was similar to the finding in Ethiopia where *Pfhrp3* negative infections were 31% a*nd Pfhrp2* negative infections 27% from 610 febrile individuals presenting to health facilities (37). The analysis of whole genome sequencing data suggested that the *Pfhrp3* deletions were older and independently arose multiple times, while *Pfhrp2* deletions are under more recent strong selective pressure (37).

Currently, in the Horn of Africa, Ethiopia (19, 37) and Eritrea (16, 38), there has been a switch from HRP2-based to LDH-based RDTs, due to the countries being above the recommended WHO *Pfhrp2/3* gene deletion prevalence threshold of 5%. The increasing data for *Pfhrp2/3* deletions in Africa, though at a low frequency, calls for continued molecular surveillance of these genetic markers in a region that is heavily reliant on RDTs for malaria diagnosis and downstream case management.

## Data Availability

Original datasets are available in a publicly accessible repository: The original contributions presented in the study are publicly available. This data can be found here: https://doi.org/10.5281/zenodo.7594474
