# Supplementary material for: Low frequency of Plasmodium falciparum hrp2/3 deletions from symptomatic infections at a primary healthcare facility in Kilifi, Kenya
Source: Front Epidemiol. 2023 Feb 21;3:1083114. doi: 10.3389/fepid.2023.1083114 (PMC10910971; doi:10.3389/fepid.2023.1083114)
Supplement: Supplementary file 1 [file Table1.docx]

**Supplementary material**

Supplementary Table 1. Target genes, their primers, and annealing temperatures

| Gene name | Gene ID | Size of gene (bp) | Target size (bp) | Primers | Annealing temperatures (°C) |
| --- | --- | --- | --- | --- | --- |
| *hrp2* | PF3D7_0831800 | 3777 | 816 | F1- 5′-CAAAAGGACTTAATTTAAATA AGAG-3′  F2- 5′ATTATTACACGAAACTCAAGCAC-3′  R1- 5′-AATAAATTTAATGGCGTA GGCA-3′ | 54 |
| *hrp3* | PF3D7_1372200 | 2605 | 719 | F1- 5′-AAT GCAAAAGGACTTAATTC-3′  F2 - 5′-AAATAAGAGATTATTACACGAAAG-3′  R1 - 5′-TGG TGTAAGTGATGCGTAGT-3′ | 54 |
| *dhfr* | PF3D7_0417200 | 2169 | 508 | F-5’-GAACAAGTCTGCGACGTTTTC-3’  R -5’-CTTGATAAACAACGGAACCTCC-3’ | 58 |
| **18S* rDNA | PF3D7_1148600 | 2150 | 133 | F-5’- GTAATTGGAATGATAGGAATTTACAAGGT-3’  R-5’-TCAACTACGAACGTTTTAACTGCAAC-3’ | 60 |

bp-base pairs. The gene marked with an asterix (*) was targeted using the RT-PCR based probe

Supplementary Table 2. Tandem amino acid repeat types in Pfhrp2 and Pfhrp3

| **Repeat Type** | **Sequence** | ***hrp2*** | ***hrp3*** |
| --- | --- | --- | --- |
| 1 | AHHAHHVAD | 🗸 | × |
| 2 | AHHAHHAAD | 🗸 | × |
| 3 | AHHAHHAAY | 🗸 | × |
| 4 | AHH | 🗸 | 🗸 |
| 5 | AHHAHHASD | 🗸 | × |
| 6 | AHHATD | 🗸 | × |
| 7 | AHHAAD | 🗸 | 🗸 |
| 8 | AHHAAY | 🗸 | × |
| 9 | AAY | 🗸 | × |
| 10 | AHHAAAHHATD | 🗸 | × |
| 15 | AHHAHHAAN | × | × |
| 16 | AHHAAN | × | 🗸 |
| 17 | AHHDG | × | 🗸 |
| 18 | AHHDD | × | 🗸 |
| 19 | AHHAA | × | 🗸 |
| 20 | SHHDD | × | 🗸 |

🗸- present, ×- absent
